# Prevalence of and Time to Suboptimal Treatment Patterns Among People with HIV on Antiretroviral Therapy in the United States

**DOI:** 10.1007/s10461-025-05021-1

**Published:** 2026-02-02

**Authors:** Travis Lim, Paul McDwyer, Woodie Zachry, Mary J. Christoph, Amy R. Weinberg

**Affiliations:** Gilead Sciences, Inc., 333 Lakeside Dr, Foster City, CA 94404 USA

**Keywords:** Adherence, Antiretroviral therapy, HIV, Persistence, Switching, Adherencia, Terapia antirretroviral, VIH, Persistencia, Cambio

## Abstract

Modern antiretroviral therapy (ART) regimens have increased life expectancy and improved quality of life in people with HIV (PWH). However, the necessity for lifelong ART use presents adherence and persistence challenges. Recent population-level data regarding such challenges with contemporary daily oral ART regimens are limited, particularly in the United States. This observational, retrospective, noncomparative cohort study used data from HealthVerity MarketPlace closed medical and pharmacy claims from PWH ≥ 18 years of age (treatment naive or treatment experienced) who were insured in the United States. Eligible PWH had ≥ 2 pharmacy refills for a Department of Health and Human Services (DHHS) guideline-recommended complete daily oral ART regimen between January 1, 2016, and November 30, 2023, with ≥ 365 days of continuous baseline enrollment and ≥ 180 days of follow-up. The index date was the date of the first recorded pharmacy claim for a complete ART regimen, regardless of prior treatment experience. Rolling adherence was measured using the Continuous, Multiple Interval Measure of Medication Acquisition method that compared the timing of prescription fills versus the number of days the prescription was intended to last, within blocks of 90 days. Suboptimal adherence was defined as proportion of days covered < 85%. Treatment interruption was defined as a gap in ART medication supply of > 90 days, followed by resumption of ART at any point, with combined treatment interruption/discontinuation defined as a > 90-day gap in ART medication supply, regardless of whether ART was restarted. Switching between DHHS guideline-recommended ART regimens and from DHHS guideline-recommended to nonguideline-recommended ART regimens was also evaluated. A total of 73,533 PWH were included in the study (60,062 [81.7%] treatment naive and 13,471 [18.3%] treatment experienced). The proportion of PWH on treatment for ≥ 1 year who remained adherent during all 90-day blocks decreased from 40.2% (95% confidence interval [CI], 39.6%–40.8%) by the end of Year 1, to 24.2% (95% CI, 23.7%–24.6%) at the end of Year 2, and to 17.7% (95% CI, 17.3%–18.1%) at the end of Year 3 when standardized by age group-sex-region. The standardized annual prevalence of suboptimal adherence remained relatively constant between 2017 and 2022 (46.7%–53.2%). The standardized proportion of PWH who persisted on daily oral ART without treatment interruption was 81.2% (95% CI, 80.8%–81.6%) after 1 year, 74.1% (95% CI, 73.7%–74.6%) after 2 years, and 70.6% (95% CI, 70.1%–71.1%) after 3 years. Switching between DHHS guideline-recommended daily oral ART regimens remained below 10% between 1 and 3 years of follow-up and was similar for switches from guideline-recommended to nonguideline-recommended daily oral ART regimens. The findings of this study suggest that suboptimal adherence to daily oral ART remains a challenge, even with the availability of modern regimens.

## Resumen

Los regímenes modernos de terapia antirretroviral (TAR) han aumentado la esperanza de vida y mejorado la calidad de vida de las personas con VIH (PWH). Sin embargo, la necesidad de usar TAR de por vida presenta desafíos en cuanto a adherencia y persistencia. Los datos poblacionales recientes sobre estos desafíos con los regímenes de TAR orales diarios contemporáneos son limitados, especialmente en Estados Unidos. Este estudio de cohorte observacional, retrospectivo y no comparativo utilizó datos de reclamos médicos y farmacéuticos resueltos de HealthVerity MarketPlace de personas con VIH ≥ 18 años (con o sin experiencia en tratamiento) aseguradas en Estados Unidos. Las personas elegibles recibieron ≥ 2 resurtidos de farmacia para un régimen completo de TAR oral diario recomendado por las directrices del Departamento de Salud y Servicios Humanos (DHHS) entre el 1 de enero de 2016 y el 30 de noviembre de 2023, con ≥ 365 días de inscripción inicial continua y ≥ 180 días de seguimiento. La fecha índice fue la fecha de la primera solicitud de reembolso de farmacia registrada para un régimen completo de TAR, independientemente de la experiencia previa con el tratamiento. La adherencia continua se midió mediante el método de Medición Continua de Intervalos Múltiples de Adquisición de Medicamentos, que comparó el momento de surtir la receta con el número de días de duración prevista, en bloques de 90 días. La adherencia subóptima se definió como una proporción de días cubiertos < 85%. La interrupción del tratamiento se definió como una interrupción en el suministro de medicamentos para el TAR de más de 90 días, seguida de su reanudación en cualquier momento. La interrupción/suspensión combinada del tratamiento se definió como una interrupción en el suministro de medicamentos para el TAR de más de 90 días, independientemente de si se reinició o no. También se evaluó el cambio entre regímenes de TAR recomendados por las directrices del DHHS y de regímenes de TAR recomendados por las directrices del DHHS a regímenes de TAR no recomendados. Se incluyó en el estudio a un total de 73.533 personas con VIH (60.062 [81,7%] sin tratamiento previo y 13.471 [18,3%] con tratamiento previo). La proporción de personas con VIH en tratamiento durante ≥ 1 año que mantuvieron la adherencia durante todos los bloques de 90 días disminuyó del 40,2% (intervalo de confianza [IC] del 95%: 39,6% − 40,8%) al final del primer año al 24,2% (IC del 95%: 23,7% − 24,6%) al final del segundo año y al 17,7% (IC del 95%: 17,3% − 18,1%) al final del tercer año, al estandarizarse por grupo de edad, sexo y región. La prevalencia anual estandarizada de adherencia subóptima se mantuvo relativamente constante entre 2017 y 2022 (46,7%-53,2%). La proporción estandarizada de personas con VIH que persistieron con TAR oral diaria sin interrupción del tratamiento fue del 81,2% (IC del 95%, 80,8% − 81,6%) después de 1 año, del 74,1% (IC del 95%, 73,7% − 74,6%) después de 2 años y del 70,6% (IC del 95%, 70,1% − 71,1%) después de 3 años. El cambio entre los regímenes de TAR oral diario recomendados por las directrices del DHHS se mantuvo por debajo del 10% entre 1 y 3 años de seguimiento, y fue similar para los cambios de regímenes de TAR oral diario recomendados por las directrices a regímenes no recomendados por ellas. Los hallazgos de este estudio sugieren que la adherencia subóptima al TAR oral diario sigue siendo un desafío, incluso con la disponibilidad de regímenes modernos.

## Introduction

As research continues toward ending the HIV epidemic, the introduction of antiretroviral therapy (ART) has resulted in a substantial decrease in HIV-related deaths in the United States from a peak of approximately 50,000 in 1995 to fewer than 4800 in 2023 [1, 2]. With current guideline-recommended ART regimens, people with HIV (PWH) may have a life expectancy almost equivalent to that of the general population [3, 4]. It is well established that suboptimal ART treatment patterns, including treatment interruptions, discontinuations, and adherence challenges, can result in the development of drug-resistant mutations [5], virologic failure [6, 7], poorer physical and mental health [8], forward transmission [9], increased pressure on the health care system [10], and mortality [11]. Thus, adequate HIV viral suppression [12–16] is largely achieved through treatment adherence and persistence (i.e., taking ART consistently as prescribed) [17, 18]. As such, ART treatment of PWH constitutes the second of 4 pillars (i.e., diagnose, treat, prevent, and respond) of the US Centers for Disease Control and Prevention (CDC) strategy to End the HIV Epidemic in the United States [19]. According to the CDC, up to 769,575 PWH in the United States received some form of medical care during 2022 [20]. However, even with modern, simplified daily oral ART regimens, including once-daily single-tablet formulations, suboptimal treatment patterns continue to be a challenge for some PWH [8, 21–23], and new HIV infections in the United States continue to occur [24]. Understanding the current patterns of suboptimal treatment in PWH in the United States can inform targeted interventions across the CDC’s pillars to End the HIV Epidemic.

Herein, we provide an update to existing literature regarding observations of daily oral ART adherence and persistence, which to date have focused on the period up to 2019 [8, 22, 23, 25, 26]. This study includes the use of all contemporary US Department of Health and Human Services (DHHS) guideline-recommended daily oral ART regimens within a large US commercial claims database between 2017 and 2024 (as of May 2024). A time-to-event analysis was also conducted to better understand when PWH may be at the greatest risk for suboptimal treatment behaviors during the course of their treatment journey.

## Methods

### Study Design

This was an observational, retrospective, noncomparative cohort study conducted using HealthVerity MarketPlace closed medical and pharmacy claims from de-identified individuals insured in the United States under commercial health insurance, Medicare Advantage, or Medicaid plans. The study population was PWH who had been prescribed ART between January 1, 2016, and May 31, 2024. Eligible PWH were ≥ 18 years of age at the first ART claim, had a medical claim for an HIV diagnosis (*International Classification of Diseases, Tenth Revision* [ICD-10] codes: B20*, Z21*, V08, R75, O98.71*, and *ICD, Ninth Revision* [ICD-9] codes: 042 and 79571) at any point during the study period, were enrolled in a pharmacy benefit plan during the pharmacy claims period, and had ≥ 2 consecutive refills comprising any complete daily oral ART regimen during the study period. PWH who were included in analyses of adherence, treatment interruption, or regimen switching must have had ≥ 365 days of continuous baseline enrollment immediately prior to the index date and ≥ 180 days of follow-up starting from the index date. The index date was the date of the first recorded pharmacy claim in the study period for a complete regimen of a DHHS guideline-recommended daily oral ART, regardless of prior treatment experience. DHHS guidelines for recommended ART evolved over the study period; therefore, the current guidelines at that time were evaluated for each individual ART claim to determine if the regimen was considered a guideline-recommended initial regimen for most people. For those who switched regimens, DHHS guidelines were again evaluated to determine if switching was from recommended-to-recommended regimen or from recommended-to-nonrecommended regimen. PWH were considered treatment naive if they had no ART claim ever before the index regimen. PWH were considered treatment experienced if they had ≥ 1 ART claim at any point prior to the index date. PWH were excluded if they were on ART regimens other than a complete daily oral medication. The study entailed secondary analysis of existing claims data; therefore, informed consent was not obtained.

### Outcome: ART Adherence

To increase the time resolution of adherence, a novel rolling adherence measure was constructed. To achieve this, follow-up time was subdivided into contiguous blocks of 90 days starting from the index date (i.e., time 0). The Continuous, Multiple Interval Measure of Medication Acquisition (CMA7) method was applied by comparing the timing of prescription fills versus the number of days the prescription was supposed to last within those 90 days [27, 28], allowing excess supply to carry over into the subsequent 90-day block. Early refills were allowed to carry forward ≤ 7 days of coverage and could be accumulated, with a maximum carryover of 7 days. Suboptimal adherence was defined using a threshold of < 85% based on recent studies of INSTI forgiveness, with a sensitivity analysis performed using a threshold of < 75% [29, 30]. Thus, suboptimal adherence was the day at which ≥ 14 days without medication supply had accumulated within any 90-day block. Two adherence measure outcomes were examined: time from index to first fall below 85% rolling adherence and annual prevalence of suboptimal adherence. For time-to-event analysis, Kaplan-Meier plots of rolling adherence were constructed, censoring at insurance disenrollment or on the last day of medication supply upon discontinuing therapy altogether with no further ART claims, or if the PWH reached the end of the study period and were still enrolled. Annual prevalence was calculated as the number of PWH who had ≥ 1 rolling block of < 85% adherence divided by enrolled person-years; prevalence was restricted to PWH who had a full year of enrollment of any given year.

### Outcome: ART Treatment Interruption and Discontinuation

Treatment interruption was defined as a gap in ART medication supply > 90 days, followed by resumption of ART at any point; the minimum follow-up time to be included in an analysis of this outcome was 270 days (90 days to establish ≥ 2 fills of the index regimen, ≥ 90 days of treatment interruption, and then 90 days to check for evidence of resuming any ART). A combined treatment interruption/discontinuation outcome was defined as a gap in ART medication supply > 90 days, regardless of whether ART was resumed. For time-to-event analysis, Kaplan-Meier plots were constructed. For treatment interruption, PWH were followed until the start of a 90-day gap in ART with eventual resumption, censored at the start of a 90-day gap if they never restarted, or censored at disenrollment. For treatment interruption/discontinuation, PWH were followed as persistent until the start of a ≥ 90-day gap and were censored at disenrollment. Annual prevalence was calculated as the number of PWH who had ≥ 1 treatment interruption or combined treatment interruption/discontinuation divided by enrolled person-years.

### Outcome: ART Switching

Two criteria were used to evaluate treatment switch: (1) prescription for a DHHS guideline-recommended regimen that differed from the index regimen (referred to as a “guideline-to-guideline switch”) and (2) prescription for any ART that was not a guideline-recommended regimen and was not part of the index regimen (referred to as “guideline-to-nonguideline switch”). To assess whether regimens were part of recommended guidelines, the contemporaneous *DHHS Guidelines for the Use of Antiretroviral Agents in Adults and Adolescents Living with HIV* available at the time of the prescription were reviewed. Guideline regimens were any of those categorized as “recommended,” “recommended for most people,” “recommended in certain clinical situations,” or “alternative.” PWH must have been on the index regimen and the new regimen for 90 days each and have ≥ 2 fills of each; an intervening treatment interruption was permitted. Time from initiation of index regimen to first treatment switch event was assessed using Kaplan-Meier analysis, censoring at disenrollment or discontinuation of ART. Annual prevalence was calculated as the number of PWH who switched from the index regimen divided by enrolled person-years. A person could contribute a maximum of 1 switch per type (i.e., guideline-to-guideline switch or guideline-to-nonguideline switch) per year.

### Statistical Analysis

Prevalence and time-to-event outcomes were calculated as percentages with corresponding 95% confidence intervals (CIs). For time-to-event outcomes, entry dates for PWH were staggered across different periods but were centered at time zero for analysis purposes, regardless of the actual calendar date of entry. For prevalence outcomes, PWH were not required to contribute data across all study years but must have had a complete year of follow-up to be included in the analysis for any given year. Therefore, different populations may have contributed to each year depending on the date of entry and censoring of individual PWH. To improve the generalizability of the results, direct standardization was applied to compensate for any differences between the study sample in the claims databases and a standard population as described [31]. The standard population of PWH was generated using estimates released by the CDC National Center for HIV, Viral Hepatitis, Sexually Transmitted Diseases, and Tuberculosis Prevention of known demographic and clinical characteristics of PWH receiving any care, which were available at the level of disaggregation (e.g., by age, sex, and region) between 2017 and 2022 [20, 24]. To standardize annual prevalence rates, age group-sex-region-year–stratified versions of each rate were generated and applied to the CDC estimates, stratified by the same age group-sex-region for each year. These results were then summed by year to get overall rates. To standardize time-to-event data, age group-sex-region-year–stratified proportions of PWH who remained enrolled without the event of interest from the index date until the end of the first, second, and third year of follow-up were generated based on Kaplan-Meier plots for 2017 to 2022. Since time-to-event rates refer to an index date that could occur at any time during the study period, the stratified proportions were applied to the corresponding stratum average of the CDC standard population of PWH who received any care across 2017 to 2022 for each combination of age group-sex-region. Baseline characteristics were compared using chi-squared tests for categorical variables and the Mann-Whitney U test for age (Kolmogorov-Smirnov test showing non-normality with D = 0.072, *P* < 0.01). For Kaplan-Meier curves, the log-rank test was used to compare the difference in the survival of treatment-experienced versus treatment-naive populations across all follow-up. This test evaluates whether the probability of remaining above 80% compliance differs over time between groups. The log-rank statistic was assessed using the chi-square distribution, and a two-sided *P* value was reported. For annual prevalence results that exhibited the possibility of a linear trend over time, we applied the Cochran-Armitage trend test of unstandardized prevalence values by year. All statistical analyses were performed using SAS v9.4 (SAS Institute, Cary, NC, USA).

## Results

### Study Population

From an initial 777,459 people with a prior HIV diagnosis at any time, a total of 73,533 (9.5%) PWH fulfilled all the inclusion criteria. The main reasons for exclusion were not having ≥ 1 pharmacy claim for any ART from January 2016 to November 2023 (*n* = 351,008) and not having 365 days of continuous enrollment pre-index and 180 days of continuous enrollment post-index (*n* = 208,811). Among included PWH, 60,062 (81.7%) were treatment naive, and 13,471 (18.3%) were treatment experienced. The mean (standard deviation [SD]) age at the ART index date was 41.9 (13.2) years and 46.1 (12.0) years for the treatment-naive and treatment-experienced PWH, respectively; up to 74.7% of all PWH were male (Table 1). The most common ART regimen was bictegravir/emtricitabine/tenofovir alafenamide in treatment-naive PWH (47.3%) and elvitegravir/cobicistat/emtricitabine/(tenofovir alafenamide or tenofovir disoproxil fumarate) in treatment-experienced PWH (30.5%; Table 2).

**Table 1:** Demographic characteristics at the antiretroviral therapy index date

	Treatment naive (*N* = 60,062)	Treatment experienced (*N* = 13,471)	All PWH (*N* = 73,533)	Treatment naive vs. treatment experienced	
				Test statistic	*P* value
Median (Q1, Q3) age, years	41 (31, 53)	48 (36, 55)	42 (32, 53)	U = 1160.7	< 0.0001
Age category, n (%)				χ^2^ = 1450.2	< 0.0001
18–24 years	5349 (8.9)	446 (3.3)	5795 (7.9)		
25–34 years	15,760 (26.2)	2394 (17.8)	18,154 (24.7)		
35–44 years	13,600 (22.6)	2784 (20.7)	16,384 (22.3)		
45–54 years	12,642 (21.0)	4213 (31.3)	16,855 (22.9)		
55–64 years	10,475 (17.4)	3048 (22.6)	13,523 (18.4)		
≥65 years	2236 (3.7)	586 (4.4)	2822 (3.8)		
Sex, n (%)				χ^2^ = 8.4	0.0037
Male	44,724 (74.5)	10,193 (75.7)	54,917 (74.7)		
Female	15,338 (25.5)	3278 (24.3)	18,616 (25.3)		
Region, n (%)				χ^2^ = 4239.3	< 0.0001
West	13,331 (22.2)	1225 (9.1)	14,556 (19.8)		
Northeast	9954 (16.6)	2,221 (16.5)	12,175 (16.6)		
South	20,044 (33.4)	3627 (26.9)	23,671 (32.2)		
Midwest	7764 (12.9)	1684 (12.5)	9448 (12.8)		
Multiple regions	1954 (3.3)	318 (2.4)	2272 (3.1)		
Missing	7015 (11.7)	4396 (32.6)	11,411 (15.5)		
Year of index date, n (%)				χ^2^ = 35,000.7	< 0.0001
2016	4285 (7.1)	10,583 (78.6)	14,868 (20.2)		
2017	4444 (7.4)	538 (4.0)	4982 (6.8)		
2018	4826 (8.0)	406 (3.0)	5232 (7.1)		
2019	7230 (12.0)	427 (3.2)	7657 (10.4)		
2020	11,154 (18.6)	570 (4.2)	11,724 (15.9)		
2021	9288 (15.5)	354 (2.6)	9642 (13.1)		
2022	11,341 (18.9)	367 (2.7)	11,708 (15.9)		
2023	7494 (12.5)	226 (1.7)	7720 (10.5)		

PWH, people with HIV; Q1, first quartile; Q3, third quartile; U, Mann Whitney U test statistic

**Table 2:** Antiretroviral regimen at the index date

ART regimen, n (%)	Treatment naive (*N* = 60,062)	Treatment experienced (*N* = 13,471)	All PWH (*N* = 73,533)	Treatment naive vs. treatment experienced	
				Test statistic	*P* value
B/F/TAF	28,405 (47.3)	844 (6.3)	29,249 (39.8)	χ^2^ = 11,376.6	< 0.0001
DTG/ABC/3TC	6288 (10.5)	2428 (18.0)	8716 (11.9)		
DTG + (TAF or TDF) + (FTC or 3TC)	4188 (7.0)	659 (4.9)	4847 (6.6)		
DTG/3TC	2381 (4.0)	96 (0.7)	2477 (3.4)		
(DRV/c or DRV/r) + (TAF or TDF) + (FTC or 3TC)	3286 (5.5)	1072 (8.0)	4358 (5.9)		
EVG/c/FTC/(TAF or TDF)	9300 (15.5)	4108 (30.5)	13,408 (18.2)		
RAL + (TAF or TDF) + (FTC or 3TC)	1262 (2.1)	864 (6.4)	2126 (2.9)		
(ATV/c or ATV/r) + (TAF or TDF) + (FTC or 3TC)	560 (0.9)	638 (4.7)	1198 (1.6)		
(DRV/c or DRV/r) + ABC/3TC	151 (0.3)	177 (1.3)	328 (0.4)		
DOR/TDF/3TC or DOR + TAF/c/FTC	665 (1.1)	52 (0.4)	717 (1.0)		
EFV + (TAF or TDF) + (FTC or 3TC)	73 (0.1)	73 (0.5)	146 (0.2)		
RPV/(TAF or TDF)/FTC	3379 (5.6)	2439 (18.1)	5818 (7.9)		
DRV/r + RAL twice daily	28 (< 0.1)	8 (0.1)	36 (< 0.1)		
DRV/r once daily + 3TC	3 (< 0.1)	3 (< 0.1)	6 (< 0.1)		
ABC/3TC/ATV/r	23 (< 0.1)	0	23 (< 0.1)		
ABC/3TC/ATV/c	6 (< 0.1)	1 (< 0.1)	7 (< 0.1)		
ABC/3TC/RAL	24 (< 0.1)	3 (< 0.1)	27 (< 0.1)		
DOR/FTC/TAF	16 (< 0.1)	1 (< 0.1)	17 (< 0.1)		
EFV/FTC/TAF	24 (< 0.1)	5 (< 0.1)	29 (< 0.1)		

3TC, lamivudine; ABC, abacavir; ART, antiretroviral therapy; ATV, atazanavir; B, bictegravir; c, cobicistat; DOR, doravirine; DRV, darunavir; DTG, dolutegravir; EFV, efavirenz; EVG, elvitegravir; F or FTC, emtricitabine; r, ritonavir; PWH, people with HIV; RAL, raltegravir; RPV, rilpivirine; TAF, tenofovir alafenamide; TDF, tenofovir disoproxil fumarate

### Adherence

The mean (SD) follow-up was 1477 (901) days, and the median time to poor adherence was 255 days. The age group-sex-region–standardized proportion of PWH on treatment for ≥ 1 year who remained adherent during all 90-day blocks (i.e., did not drop below the threshold of 85%) was 40.2% (95% CI, 39.6%−40.8%). By the end of Year 2, only 24.2% (95% CI, 23.7%−24.6%) of PWH remained fully adherent during all 90-day blocks, and this dropped further to 17.7% (95% CI, 17.3%−18.1%) by the end of Year 3.

In a sensitivity analysis using a threshold of < 75% for rolling adherence, the age group-sex-region–standardized proportion of PWH remained adherent by the end of Years 1, 2, and 3 was 50.5%, 33.8%, and 26.3%, respectively. A subgroup analysis examining age groupspecific time to nonadherence showed that PWH ≥ 55 years of age had similar proportions of suboptimal rolling adherence as the overall standard population, with 45.0%, 29.4%, and 21.9% reaching the end of Years 1, 2, and 3 without suboptimal adherence in any 90-day block, respectively.

**Fig. 1:**
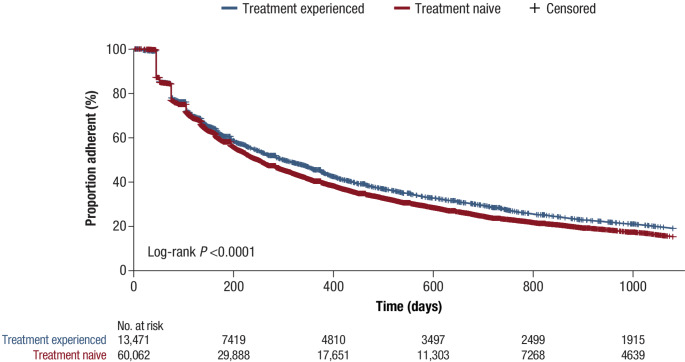
Kaplan-Meier Curve for Time to Suboptimal Adherence Within Rolling 90-Day Time Blocks in Treatment-Naive and Treatment-Experienced PWH. PWH, people with HIV

Time to suboptimal adherence was shorter in treatment-naive PWH compared with those who were treatment experienced (χ^2^ = 100.7, log-rank *P* < 0.0001; Fig. 1). Of note, Kaplan-Meier curves could not be readily standardized to a reference population, as this would require applying the standardization method across a dynamic, continuous-time proportion, which is beyond the scope of direct standardization.

**Fig. 2:**
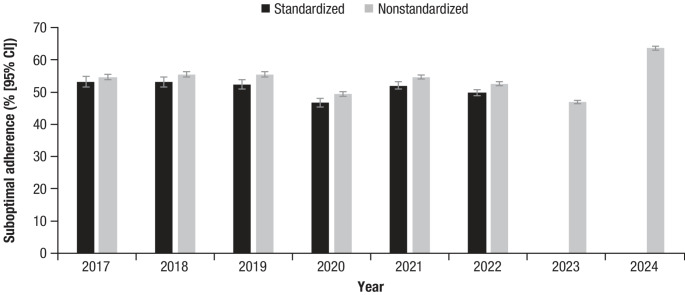
Annual Age Group-Sex-Region–Standardized and –Nonstandardized Proportion of ≥ 1 Rolling 90-Day Block of Suboptimal Adherence Among PWH on ART. ART, antiretroviral therapy; CI, confidence interval; PWH, people with HIV

For age group-sex-region–standardized trends over time, up to 53.2% of PWH per enrolled person-year of follow-up time had ≥ 1 block of suboptimal adherence. This prevalence remained consistent with a minor fluctuation from 2017 to 2022, the years when standard reference populations were available (Fig. 2). This is slightly lower and less variable than the nonstandardized proportion of PWH who had suboptimal adherence (from 2017–2024; range, 47.0%−63.7%), which was notably lower in 2020 and 2023, but higher in 2024. The prevalence of suboptimal adherence periods among the nonstandardized proportion of PWH appeared relatively stable over time, with a test of trends not reaching statistical significance (Cochran-Armitage trend test; *T* = − 1.9, *P* = 0.0593).

### Treatment Interruption and Discontinuation

The age group-sex-region–standardized proportion of PWH who persisted on ART over 1 year without treatment interruption was 81.2% (95% CI, 80.8%−81.6%). This dropped to 74.1% (95% CI, 73.7%−74.6%) by the end of 2 years and to 70.6% (95% CI, 70.1%−71.1%) by the end of 3 years. The age group-sex-region–standardized proportion of PWH who persisted on ART without a treatment interruption (regardless of whether they resumed ART or discontinued) was 69.7% (95% CI, 69.2%−70.2%) at Year 1, 54.3% (95% CI, 53.7%−54.9%) at Year 2, and 45.8% (95% CI, 45.2%−46.5%) at Year 3 after index.

**Fig. 3:**
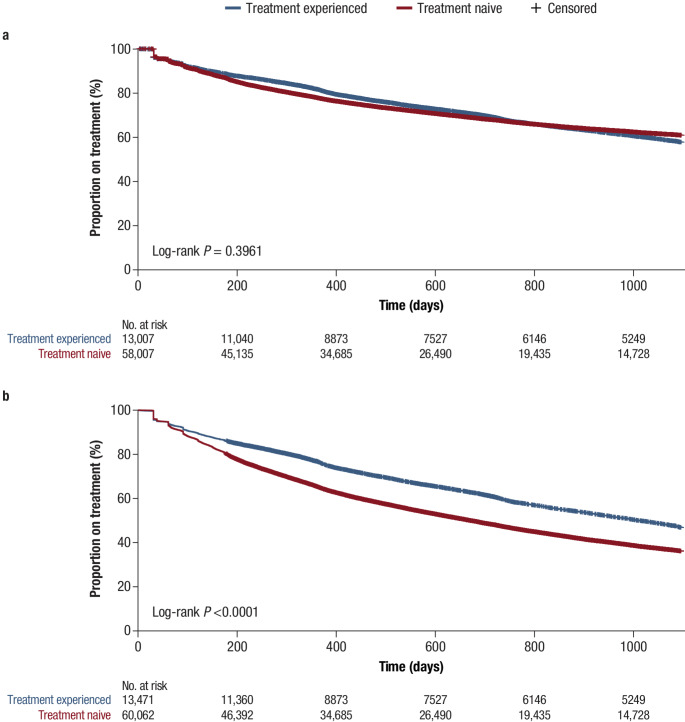
Kaplan-Meier Curve for Time to **a** Treatment Interruption and **b** Combined Treatment Interruption/Discontinuation in Treatment-Naive and Treatment-Experienced PWH. PWH, people with HIV

Median time to combined treatment interruption/discontinuation was 728 days. When comparing treatment-naive and treatment-experienced PWH, time to treatment interruption was similar between groups (χ^2^ = 0.7, log-rank *P* = 0.3961; Fig. 3a), while time to combined treatment interruption/discontinuation was shorter in those who were treatment naive (χ^2^ = 621.9, log-rank *P* < 0.0001; Fig. 3b). As noted above, Kaplan-Meier curves were not directly standardized.

**Fig. 4:**
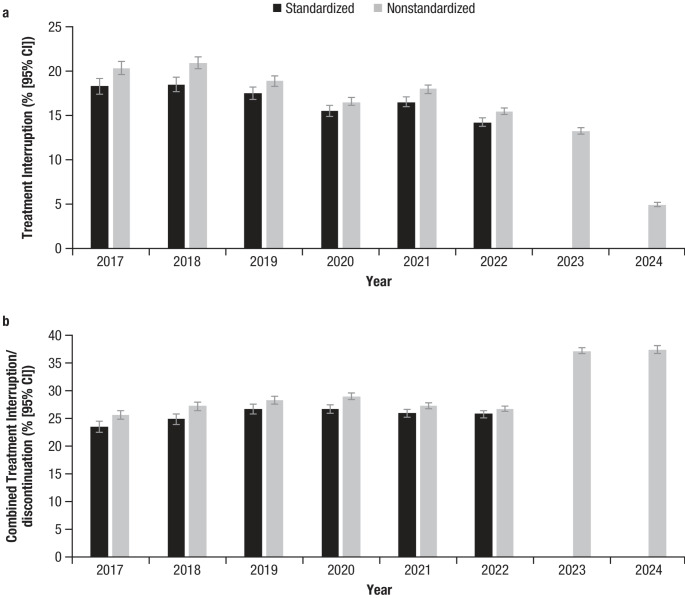
Annual Age Group-Sex-Region–Standardized and –Nonstandardized^a^ Proportion of **a** Treatment Interruption or **b** Combined Treatment Interruption/Discontinuation Among PWH on ART. ^a^At the time of the analysis, CDC standard populations were only available through 2022; thus, rates for 2023 and 2024 should be interpreted with caution. ART, antiretroviral therapy; CDC, Centers for Disease Control and Prevention; CI, confidence interval; PWH, people with HIV

The age group-sex-region–standardized trends for treatment interruption decreased slightly over time, with 18.3% (95% CI, 17.4%−19.2%) of PWH experiencing a treatment interruption per enrolled person-year of follow-up in 2017, decreasing to 14.2% (95% CI, 13.8%−14.7%) in 2022. While these trends were not formally tested, it should be noted that CIs do not overlap as the prevalence of treatment interruption decreased (Fig. 4a). From 2017 to 2022, trends were similar in the nonstandardized proportion of PWH who had a treatment interruption, with a noticeable decrease in 2023. Overall, treatment interruption became less common over time in the nonstandardized proportion of PWH, with the declining trend reaching statistical significance (Cochran-Armitage trend test; *T* = –39.9, *P* < 0.0001).

When examining age group-sex-region–standardized time trends for combined treatment interruption/discontinuation, approximately one-quarter of PWH (range, 23.4%−26.6%) per enrolled person-year of follow-up time either interrupted treatment or discontinued in each year from 2017 to 2022, with no discernable consistent trend over time (Fig. 4b). These results were slightly lower than the nonstandardized proportion of PWH with a combined treatment interruption/discontinuation (range, 25.6%−28.9% from 20172022). The nonstandardized proportion of PWH with a combined treatment interruption/discontinuation was higher in 2023 and 2024. While the prevalence of combined treatment interruption/discontinuation among the nonstandardized proportion of PWH appeared relatively stable from 2016 to 2022, a test of trends was not conducted due to high outliers that caused non-linearity in 2023 and 2024.

### Switching ART Regimens

**Fig. 5:**
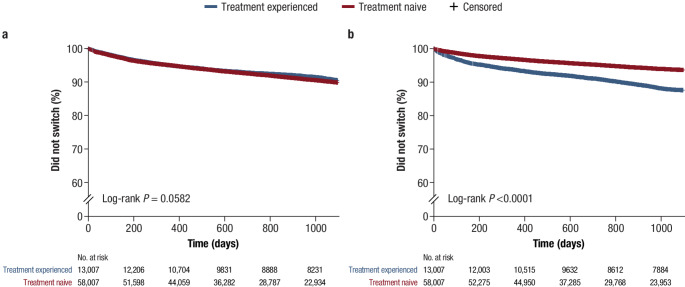
Kaplan-Meier Curve for Time to Switching From a DHHS Guideline-Recommended ART to **a** Another Guideline-Recommended ART or **b** Any Nonguideline-Recommended ART in Treatment-Naive and Treatment-Experienced PWH. ART, antiretroviral therapy; DHHS, US Department of Health and Human Services; PWH, people with HIV

Kaplan-Meier curves for time to switching from a DHHS guideline-recommended regimen to another guideline-recommended regimen were similar for treatment-naive and treatment-experienced PWH (χ^2^ = 3.6, log-rank *P* = 0.0582; Fig. 5a), while time to switching to a nonguideline-recommended regimen was shorter for treatment-experienced PWH (χ^2^ = 483.9, log-rank *P* < 0.0001; Fig. 5b). The age group-sex-region–standardized rates of switching were slightly higher between guideline-recommended regimens than from guideline-recommended regimens to nonguideline-recommended regimens. A total of 5.0% (95% CI, 4.8%−5.2%) of PWH switched between guideline-recommended regimens within the first year of follow-up post index, which increased slightly to 7.0% (95% CI, 6.8%−7.3%) and 8.3% (95% CI, 8.1%−8.6%) by the end of the second and third years of follow-up, respectively. After applying age group-sex-region standardization, switching to nonguideline-recommended regimens occurred in 4.0% (95% CI, 3.8%−4.1%) of PWH within the first year, rose to 5.4% (95% CI, 5.2%−5.7%) by the end of the second year, and rose to 6.5% (95% CI, 6.2%−6.7%) by the end of the third year.

**Fig. 6:**
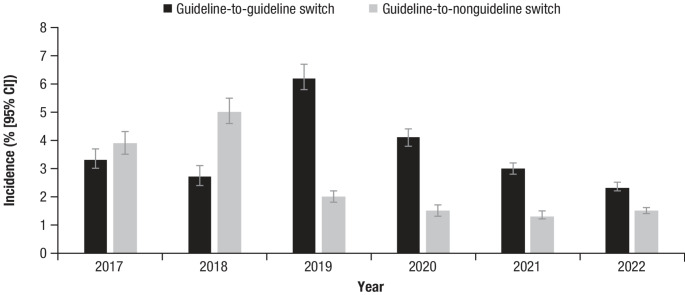
Annual Age Group-Sex-Region–Standardized Incidence of PWH Switching Between DHHS Guideline-Recommended ARTs or From a Guideline-Recommended to Any Nonguideline-Recommended ART. ART, antiretroviral therapy; CI, confidence interval; DHHS, US Department of Health and Human Services; PWH, people with HIV

The age group-sex-region–standardized incidence of switching between guideline-recommended regimens fluctuated by year (range, 2.3%−6.2%; Fig. 6), with the highest annual prevalence of switching occurring in 2019 with 6.2 people switching per 100 enrolled person-years of follow-up time. The annual prevalence of switching to a nonguideline-recommended regimen was slightly lower (range, 1.3%−5.0%), and it was highest in 2018. Since no apparent linear nor higher-order trends were observed, a trend analysis was not performed.

## Discussion

This study provides insight into several common suboptimal treatment patterns experienced by PWH taking daily oral modern ART in the United States between 2016 and 2024. Suboptimal adherence, treatment interruption, and switching regimens may result from a variety of factors, including ART side effects, tolerability issues, loss of viral suppression, and drug resistance [8, 32–36]. Herein, most PWH demonstrated periods of suboptimal adherence to daily oral ART when taken for > 1 year. Treatment interruptions were also relatively common (observed in up to 18.8% of PWH by the end of 1 year, rising to 29.4% by the end of 3 years). To our knowledge, this is the first study spanning the modern ART era with broad coverage of the US population of PWH using age group-sex-region standardization methods to improve generalizability and develop more granular adherence measures.

Overall, the prevalence of suboptimal adherence tended to be higher among treatment-naive PWH compared with treatment-experienced PWH. However, the annual prevalence of PWH with suboptimal adherence remained around 50% in both subgroups between 2017 and 2023, indicating no apparent trend over time. The annual incidence of treatment interruption was also higher in treatment-naive PWH compared with treatment-experienced PWH throughout the study period.

There is little published data assessing ART adherence according to treatment experience. One previously reported US-based study showed a higher proportion of treatment-naive PWH had > 90% adherence based on pharmacy refill records to multitablet regimens compared with treatment-experienced PWH, although the reverse was true for single-tablet regimens [37]. A separate study conducted in Cameroon reported a significantly lower risk of suboptimal adherence in participants who had been on ART for ≥ 10 years compared with those who had been on ART for < 1 year [38]. A further study using Medicare claims data found that younger age was associated with lower adherence relative to older age, which could serve as a proxy for treatment experience [26]. A potential reason for these findings may be the sustained inclusion of treatment-experienced PWH in the dataset over time. Furthermore, it should be considered that treatment-naive PWH were more likely to have initiated treatment with modern ART regimens, which have been shown to have greater adherence and persistence due to factors such as a lower pill burden [39].

Data related to switching ART regimens in US populations are also limited. The results of our study showed that switching between ART regimens was uncommon, occurring in ≤ 10% of PWH. In an analysis of claims data from 2006 to 2011, 12% of commercially insured PWH and 14% of Medicare-insured PWH had an ART switch [40]. A large global epidemiologic study from 2003 to 2014 showed that 10 years after initiating ART, 90% of treatment-naive women with HIV and 75% of treatment-naive men with HIV had a major change (e.g., switching or treatment interruption) in their ART regimen [38]. In an analysis of the Multicenter AIDS Cohort Study of men who have sex with men, the proportion who switched ART regimens during 2008 to 2017 ranged from 6% to 27%, depending on the initial regimen, with a higher percentage switching from older regimens rather than from modern regimens [33]. Notably, during the current study period, several single-tablet regimens were approved for the treatment of HIV-1, including bictegravir/emtricitabine/tenofovir alafenamide and dolutegravir/lamivudine in 2018 and 2019, respectively [41, 42]. Herein, the highest frequency of switching was observed between guideline-recommended regimens during 2019. These findings, together with the results of previous studies, may represent a lower need to switch with the newer regimens. Indeed, a recent Japanese study found that between 2019 and 2022, of 952 PWH on bictegravir/emtricitabine/tenofovir alafenamide, only 1 switched to another ART regimen [43].

Previous US claims–based studies (collecting data between 20112019) have reported ART nonadherence rates between 31% and 95% [8, 22, 23, 25, 26, 44]. However, these have used varying definitions of nonadherence (e.g., PWH self-reporting, different thresholds for proportion of days covered, and different time periods) that make comparison with the findings from the present study difficult. In general, studies that measured adherence via pharmacy claimsbased proportion of days covered tended to produce higher estimates of suboptimal or poor adherence (47%−95%; PDC < 90%−95%) [22, 23, 25, 26, 44], while only 24% of PWH reported suboptimal adherence based on missed-dose recall in a self-reported survey study [8]. The prevalence of suboptimal adherence observed in the current study aligns with that reported in several other pharmacy claimsbased studies using proportion of days covered [22, 23, 26, 44].

A strength of the study was its use of a novel method of calculating rolling adherence. Previous ART studies have examined the total proportion of days covered by medication over a complete follow-up duration. Such conventional methods could average out short periods of nonadherence, thus lacking sensitivity to true nonadherence. Conversely, the rolling adherence approach utilized herein increased the granularity/resolution of adherence over time and captured shorter periods that could have clinical significance (i.e., emergence of resistance, viral rebound, HIV transmission, etc.). Indeed, the results show that more than half of PWH had < 85% adherence at some point during the first year, which is higher than many other estimates of overall nonadherence in the literature.

An additional strength of the study was the standardization of the results to adjust the findings to what the results would have been if the study population resembled a standard population (per CDC estimates), at least in terms of observable characteristics. The minor differences observed after standardization suggest that PWH on ART in the HealthVerity database resemble the broader population of PWH on ART in the United States, supporting the generalizability of the data. At the time of the analysis, CDC standard populations were only available through 2022; thus, nonstandardized rates for 2023 and 2024 should be interpreted with caution. Indeed, we noted that the raw rate of treatment discontinuation was a high outlier in 2024.

A final key strength of this study is the use of the HealthVerity database, a large claims repository covering a substantial proportion of PWH in the United States, which yielded a study population of > 73,000 PWH. However, it should be noted that the substantial number of PWH included in the study population likely contributed to the statistically significant differences observed. Yet, such significance may not represent clinically or scientifically meaningful relevance, as even small differences can reach statistical significance in large datasets.

A limitation of the study was that an analysis of claims data cannot account for medications received outside of this system (e.g., sampling, cash payments, or through charitable organizations). Another limitation was the inability to standardize rates using race and ethnicity, which were not available at the patient level in HealthVerity, despite being reported in CDC statistical reports [20, 24].

## Conclusions

These findings confirm that adherence and persistence with daily oral ART are ongoing challenges to treatment success and public health in the United States, particularly among PWH who are treatment naive. Short, but potentially clinically meaningful, periods of poor adherence were observed. While further development of long-acting ART may help improve adherence, these data suggest that additional efforts are still needed to help PWH maintain their recommended regimen. By implementing innovative strategies, health care systems may support the ability of PWH to remain engaged in care and adherent to daily oral treatment. These results also suggest that using ART regimens with a high barrier to resistance and simplified administration schedules should be considered to afford greater forgiveness for individuals with adherence challenges.

## Data Availability

The dataset generated during the current study is not publicly available as it is a proprietary dataset.

## Declarations

### **Medical Writing, Editorial, and Other Assistance**

Medical writing and editorial support were provided by Michelle Preston, PhD, of Lumanity Communications Inc. (Yardley, PA, USA), and funded by Gilead Sciences, Inc.
